# Expansion and characterization of bone marrow derived human mesenchymal stromal cells in serum-free conditions

**DOI:** 10.1038/s41598-021-83088-1

**Published:** 2021-02-09

**Authors:** Samatha Bhat, Pachaiyappan Viswanathan, Shashank Chandanala, S. Jyothi Prasanna, Raviraja N. Seetharam

**Affiliations:** 1 Stempeutics Research Pvt Ltd, Shirdi Sai Baba Cancer Hospital, Manipal, Karnataka 576104 India; 2 Manipal Institute of Regenerative Medicine, Manipal Academy of Higher Education, Allalsandra, Yelahanka, Bengaluru, Karnataka 560065 India

**Keywords:** Stem-cell biotechnology, Mesenchymal stem cells, Stem-cell differentiation

## Abstract

Bone marrow-derived mesenchymal stromal cells (BM-MSCs) are gaining increasing importance in the field of regenerative medicine. Although therapeutic value of MSCs is now being established through many clinical trials, issues have been raised regarding their expansion as per regulatory guidelines. Fetal bovine serum usage in cell therapy poses difficulties due to its less-defined, highly variable composition and safety issues. Hence, there is a need for transition from serum-based to serum-free media (SFM). Since SFM are cell type-specific, a precise analysis of the properties of MSCs cultured in SFM is required to determine the most suitable one. Six different commercially available low serum/SFM with two different seeding densities were evaluated to explore their ability to support the growth and expansion of BM-MSCs and assess the characteristics of BM-MSCs cultured in these media. Except for one of the SFM, all other media tested supported the growth of BM-MSCs at a low seeding density. No significant differences were observed in the expression of MSC specific markers among the various media tested. In contrary, the population doubling time, cell yield, potency, colony-forming ability, differentiation potential, and immunosuppressive properties of MSCs varied with one another. We show that SFM tested supports the growth and expansion of BM-MSCs even at low seeding density and may serve as possible replacement for animal-derived serum.

## Introduction

Mesenchymal stromal cells (MSCs) have emerged as a new player in the field of regenerative medicine due to its tremendous therapeutic applications. MSCs are plastic adherent, fibroblast-like cells which are mesodermal in origin, positively expressing cell surface markers CD29, CD44, CD49a-f, CD51, CD73, CD90, CD105, CD106, CD166 and Stro-1 while being negative for CD14, CD19, CD34, CD45 and HLA DR expression and exhibit tri-lineage differentiation in vitro^1^. They are multipotent cells showing self-renewal capacity and are present in multiple tissues including bone marrow, dental pulp, synovial fluid, umbilical cord, cord blood, Wharton’s jelly, limbal tissue, adipose tissue, placenta and amniotic fluid^2,3^. Clinical applications of MSCs are essentially attributed to their key biological properties which include (i) the ability to home to sites of inflammation during tissue injury when intravenously injected, (ii) the capacity to differentiate into several cell types including cartilage, muscle, bone, connective tissue and fat cells (iii) secreting various bioactive molecules thereby stimulating recovery of injured cells and (iv) suppressing inflammation and exhibiting immunomodulatory functions^4^.

Bone marrow-derived MSCs (BM-MSCs) account for ~ 0.001–0.01% of bone marrow mononuclear cells. Due to its low abundance, extensive in vitro culturing and expansion is required to obtain adequate numbers for research or clinical application^5^. The properties of BM-MSCs such as ease of isolation from BM without causing an immunological problem, ability to expand in vitro within a short period of time, bio preservation for point-of-care delivery with minimal loss of potency, and have not shown any reported serious adverse reactions during autologous or allogenic therapy makes them an important tool in regenerative medicine^6–8^. Furthermore, BM-MSCs have proven to be safe and are being widely tested in clinical trials, thus representing a powerful therapeutic paradigm for the treatment of a wide range of debilitating diseases^9^. Currently, there are more than 130 registered/ ongoing clinical trials aimed at elucidating the potential of BM-MSCs based cell therapy worldwide (www.clinicaltrials.gov).

MSCs have been currently classified as advanced therapy medicinal products (ATMPs) and have to be manufactured complying good manufacturing practices (cGMP) ensuring consistent production and quality standards in terms of safety, identity, and potency as per EU Regulations 2003/94/EC and 91/356/EEC directives^10,11^. MSCs are traditionally cultured in Dulbecco’s modified Eagle’s medium (DMEM) supplemented with animal serum, the most widely used being fetal bovine serum (FBS). FBS contains a cocktail of macromolecules and carrier proteins, cell attachment and growth factors, nutrients, hormones, and other essential biomolecules required for the growth and expansion of MSCs^12^. However, there are safety concerns regarding the use of FBS for in vitro expansion and clinical applications of MSCs. FBS is an ill-defined supplement which results in lot to lot variation due to inconsistency in terms of the quality and quantity of bioactive component^13^. The use of FBS is discouraged by regulatory agencies due to the risk of contamination with microbiological contaminants (fungi, bacteria, prions, viruses, and endotoxins) and xenogenic compounds which may influence cell behavior. Further, collection of FBS also requires the painful death of bovine fetuses which may be considered ethically inhumane and an increase in the demand for serum which may exceed the amount of maximum serum availability globally^14^. Several controversies are arising due to the lack of common standardized protocols for MSC in vitro expansion. This is crucial since culture conditions may influence the biological behavior and properties of MSCs, affecting their performance upon transplantation^15^. In light of the above-mentioned disadvantages and concerns, regulatory bodies encourage the use of xeno-free media to delineate safer and standardized protocols for the expansion of MSCs that preserve their inherent therapeutic potential^14^. Serum-free/Xeno-free media (SFM/XFM) formulations are specific and require optimization based on cell type^16^. SFM development not only accomplishes regulatory requirements but also improves the manufacturing of cells by reducing batch to batch variability, eliminating the risk of potential xeno contaminants^17^ and the need for rigorous washing of cells during formulation thereby retaining the shelf life^18^. Recent studies have focused on developing and evaluating animal SFM as an alternative to FBS employing human-derived (such as human platelet lysate) or recombinant growth supplements (such as MSC Nutristem XF, Biological Industries; StemPro MSC SFM, Invitrogen; StemMACS-MSC XF, Miltenyi Biotec) for MSC expansion. Several groups have reported the development of in-house SFM specifically for MSC growth and expansion^19–29^. However, these SFM formulations demonstrate inconsistencies in the growth promoting potential of MSCs and require detailed investigation regarding its safety for human use. Hence, there is a need to develop GMP compliant SFM for cell therapy product^30^. More recently, animal and human SFMs that are chemically defined have been developed as potential alternatives for the expansion of clinical-grade MSCs and have been reported to exhibit superior growth kinetics while retaining other MSC characteristics^27,31–34^. Further, the conditioned medium derived from MSCs cultured in serum-free conditions has shown to be a promising source for application in regenerative medicine^35^. However, MSCs exhibit variable growth on different SFM and hence these media formulations may need to be modified based on the selected donor MSCs and their tissue sources for maximizing the therapeutic benefits^16,24^. Thus, it is essential to assess the most efficient SFM/XFM for clinical-scale MSC expansion.

In this study, we have tested six different commercially available low serum/SFM/XFM for their suitability for the growth and expansion of undifferentiated BM-MSCs (Table 1) in comparison with the serum-containing in-house medium. Towards this, we assessed and compared morphological characteristics, population doubling and doubling time, cell size, expression of cell surface markers, colony-forming ability, potency in terms of vascular endothelial growth factor (VEGF) secretion, immunosuppressive and differentiation potential of BM-MSCs cultured in these low serum/SFMs/XFMs as opposed to serum-containing medium.

**Table 1:** List of commercially available low serum/serum-free/xeno-free media evaluated in the study.

Sl No	Serum-free media	Catalog No	Company
01	RoosterNourish (containing 1% FBS)- Low serum	KT-001	RoosterBio, Inc. (MD, USA)
02	RoosterNourish-MSC XF	KT-016	RoosterBio, Inc. (MD, USA)
03	StemMACS-MSC Expansion Media Kit XF	130–104-182	Miltenyi Biotec, Germany
04	PLTMax Human Platelet Lysate	SCM141	Merck, USA
05	MSC NutriStem XF Basal Medium MSC NutriStem XF Supplement Mix MSC attachment solution	05–200-1A 05–201-1U 05–752-1H	Biological Industries, USA
06	StemXVivo Serum free Human Mesenchymal Stem cell Expansion media Recombinant Human Fibronectin Protein	CCM014 1918-FN	R&D Systems, USA

## Results

### Ex-vivo expansion of BM-MSCs using different seeding densities and analysis of cell size, morphology, and proliferation kinetics

**Figure 1:**
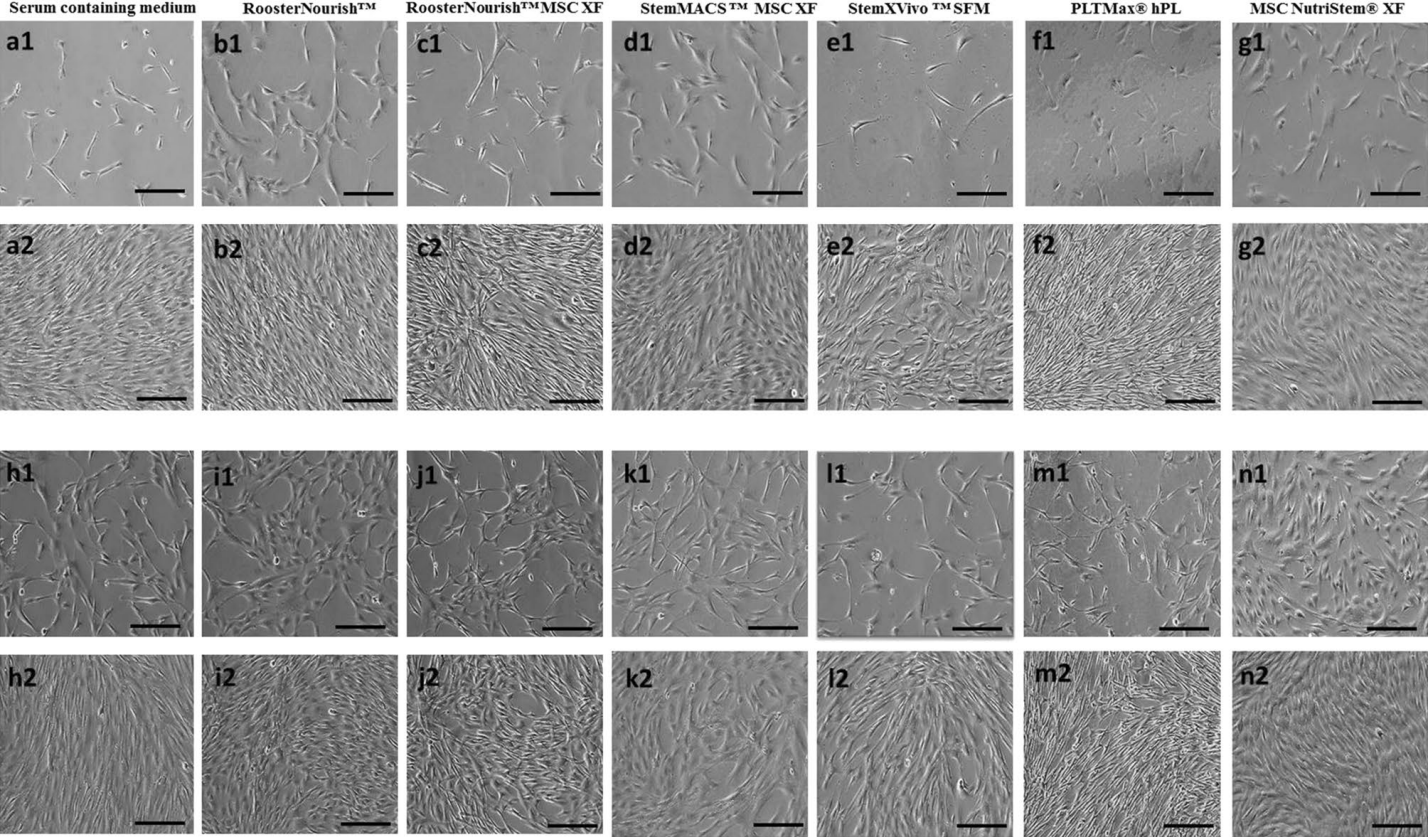
Morphology of BM-MSCs cultured at P4 stage. (**a1**, **a2**, **h1**, **h2**) BM-MSCs cultured in DMEM-KO + 10% FBS at a seeding density of 1000 cells/cm^2^ (**a1**,** a2**) and 5000 cells/ cm^2^ (**h1**, **h2**). (**a1**) Microphotographs at 10–20% confluency, a2: 80–90% confluency, (**h1**): 50–55% confluency and (**h2**): 80–90% confluency. Similarly (**b1, b2, i1, i2**) BM-MSCs cultured in RoosterNourish. **c1, c2, j1, j2**- BM-MSCs cultured in RoosterNourish-MSC XF. **d1, d2, k1, k2**- BM-MSCs cultured in StemMACS-MSC XF. (**e1, e2, i1, i2**) BM-MSCs cultured in StemXVivo serum-free media. **f1, f2, m1, m2**- BM-MSCs cultured in PLTMax human platelet lysate. (**g1, g2, n1, n2**) BM-MSCs cultured in MSC NutriStem XF . (**b1, b2, c1, c2, d1, d2, e1, e2, f1, f2, g1, g2**) MSC at a seeding density of 1000 cells/cm^2^ and (**i1, i2, j1, j2, k1, k2, l1, l2, m1, m2, n1, n2**) 5000 cells/cm^2^. (**b1, c1, d1, e1, f1, g1**) Microphotographs at 20–25% confluency. (**b2, c2, d2, e2, f2, g2**): 80–90% confluency. (**i1, j1, k1, l1, m1, n1**): 50–55% confluency and (**i2, j2, k2, l2, m2, n2**): 80–90% confluency. Experiments were performed in duplicates and the representative images are shown. The images were captured at 10× magnification with scale bar of ∼100 μm.

**Figure 2:**
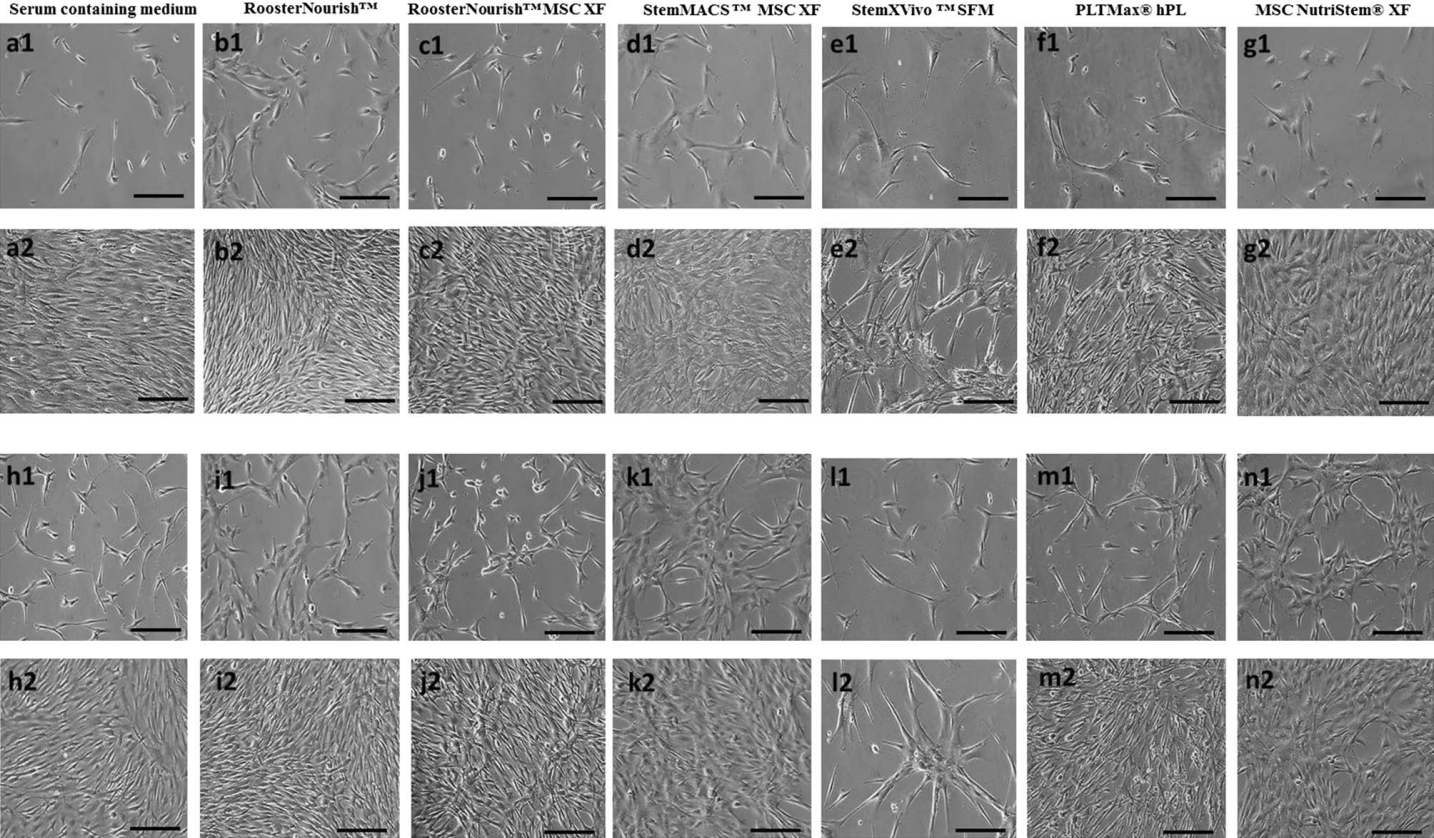
Morphology of BM-MSCs expanded at P5 stage. (**a1, a2, h1, h2**) BM-MSCs cultured in DMEM-KO + 10% FBS at a seeding density of 1000 cells/cm^2^ **(a1, a2)** and 5000 cells/cm^2^ (**h1, h2**).(**a1**) Microphotographs at 10–20% confluency, (**a2**): 80–90% confluency, **h1**: 50–55% confluency and **h2**: 80–90%confluency. Similarly (**b1, b2, i1, i2**)- BM-MSCs cultured in RoosterNourish. (**c1, c2, j1, j2**)- BM-MSCs cultured in RoosterNourish-MSC XF. **d1, d2, k1, k2**- BM-MSCs cultured in StemMACS-MSC XF. (**e1, e2, i1, i2**) BM-MSCs cultured in StemXVivo serum-free media. (**f1, f2, m1, m2**) BM-MSCs cultured in PLTMax human platelet lysate. **g1, g2, n1, n2**- BM-MSCs cultured in MSC NutriStem XF. (**b1, b2, c1, c2, d1, d2, e1, e2, f1, f2, g1, g2**)- MSC at a seeding density of 1000 cells/cm^2^ and (**i1, i2, j1, j2, k1, k2, l1, l2, m1, m2, n1, n2**) 5000 cells/cm^2^. (**b1, c1, d1, f1, g1**) Microphotographs at 20–25% confluency. (**b2, c2, d2, f2, g2**): 80–90% confluency. (**i1, j1, k1, m1, n1**): 50–55% confluency and (**i2, j2, k2, m2, n2**): 80–90% confluency. (**e1 and e2**): Microphotographs at 10–15% confluency and 40–45% confluency. **l1 and l2**: Microphotographs at 20–25% confluency and 40–50% confluency. Experiments were performed in duplicates and the representative images are shown. The images were captured at 10X magnification with scale bar of ∼100 μm.

BM-MSCs cultured in low serum/SFM/XFM at both seeding densities were well adapted to the respective media in the conditions tested and were able to proliferate linearly. At passage 4, all media supported the growth of BM-MSCs (Fig. 1); while BM-MSCs cultured in StemXVivo SFM showed less growth and proliferation at passage 5 when compared to other media (Fig. 2e2,l2). The cells grown in StemXVivo SFM at passage 5 took a long time to grow than the previous passage, which could not proliferate beyond 40% confluence. The yield of BM-MSCs cultured in StemXVivo SFM was not sufficient for cryopreservation and hence freeze–thaw analysis for immunophenotyping, differentiation, colony formation, VEGF secretion was not performed for this group; whereas for the cells seeded at 1000 cells/cm^2^, immunophenotyping was carried out post cryopreservation and MSCs cultured in other media were used for further experiments. BM-MSCs cultured in low serum/ SFM/XFM showed distinct differences in morphological characteristics, cell size, and shape. The images shown in Figs. 1 and 2 for seeding density of 1000 cells/cm^2^ were at 10–20% and 80–90% confluency, whereas for a seeding density of 5000 cells/cm^2^ were at 50–55% and 80–90% confluency respectively for both the passages. BM-MSCs cultured in RoosterNourish (containing 1% FBS) and RoosterNourish-MSC XF were spindle-shaped, elongated and showed aggregation (Fig. 1b1, b2, i1, i2 & 2 b1, b2, i1, i2). The BM-MSCs grown in StemMACS-MSC XF (Fig. 1d1, d2, k1, k2 & 2d1, d2, k1, k2) and MSC NutriStem XF (Fig. 1g1, g2, n1, n2 & 2g1, g2, n1, n2) were spindle-shaped, slender, mat-like appearance at higher confluency and were shorter and thicker when compared to control cultures. BM-MSCs cultured in PLTMax hPL (Fig. 1f1, f2, m1, m2 & 2f1, f2, m1, m2) were spindle-shaped, elongated, bright with tapering ends comparable to control cultures (Fig. 1a1, a2, h1, h2 & 2a1, a2, h1, h2) and StemXVivo MSC SFM were highly elongated, with tapering ends at P4 wherein at P5 few cells aggregated with change in shape of the cells (Fig. 1e1, e2, l1, l2 & 2e1, e2, l1, l2).

**Figure 3:**
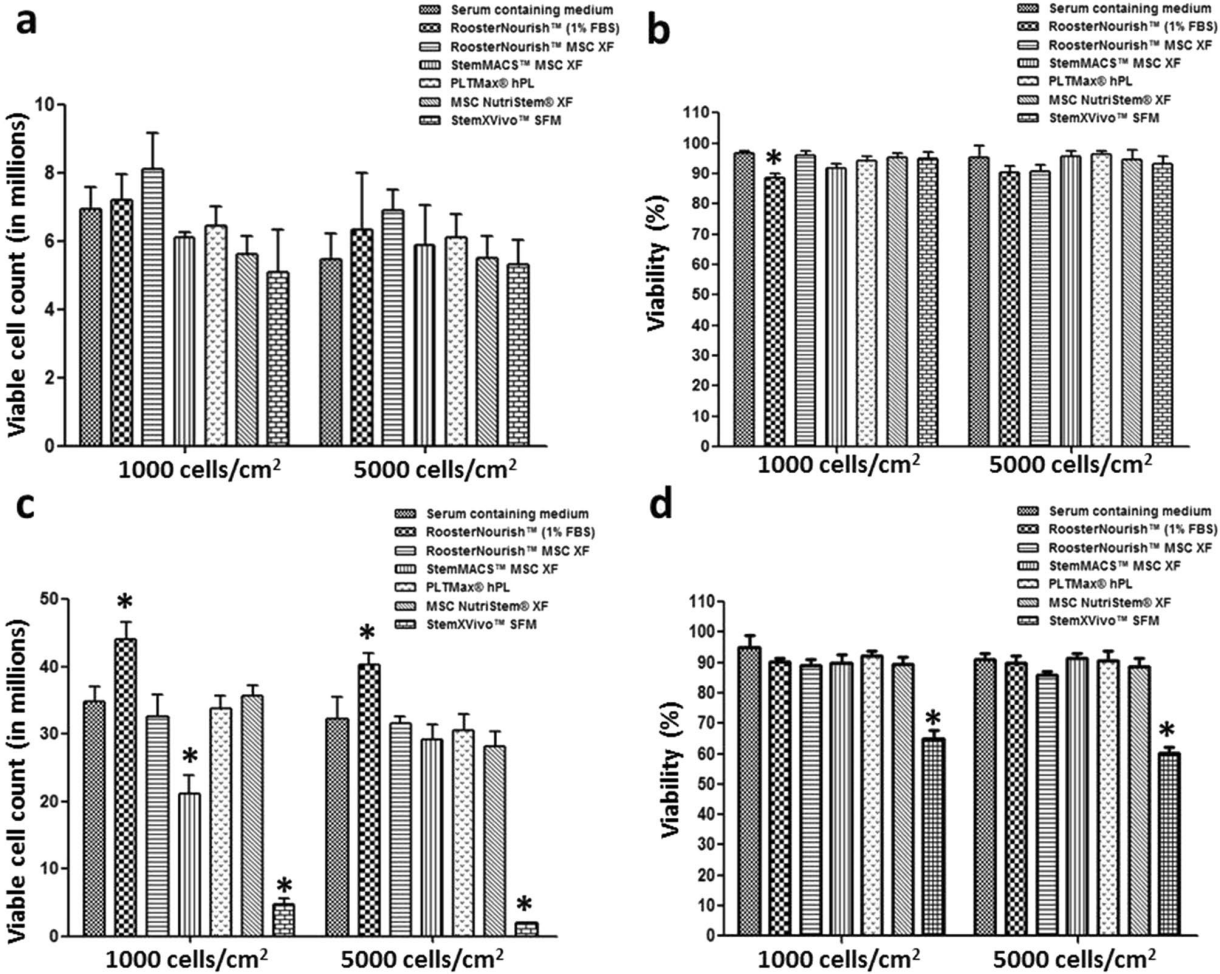
Comparison of yield and viability of BM-MSCs in six different low serum/SFM/XFM and control medium expanded at P4 **(a & b)** and P5 **(c & d)**. BM-MSCs were cultured in respective medium with two different seeding densities (1000 and 5000 cells/cm^2^). Each of the medium tested supported the growth of MSCs at P4 in both the seeding densities and the cell yield and viability was comparable with control medium **(a & b)**. Except StemXVivo SFM, all other medium tested supported the growth of MSCs in subsequent passage (P5). The yield and viability of BM-MSCs cultured in low serum/SFM/XFM was comparable with control medium at P5. **(c & d)**. Experiments were performed in duplicates. The data is represented as mean ± SD, *p < 0.05.

BM-MSCs were harvested by enzymatic digestion when the cultures attained 80–90% confluency and the cell yield and viability were assessed. The viable count of BM-MSCs cultured in low serum/SFM/XFM at P4 with seeding densities of 1000 cells/cm^2^ and 5000 cells/cm^2^ was 7.2 × 10^6^ ± 0.77 and 6.35 × 10^6^ ± 1.65 (RoosterNourish), 8.1 × 10^6^ ± 1.06 and 6.9 × 10^6^ ± 0.6 (RoosterNourish-MSC XF), 6.1 × 10^6^ ± 0.17 and 5.9 × 10^6^ ± 1.13 (StemMACS- MSC XF), 6.45 × 10^6^ ± 0.56 and 6.1 × 10^6^ ± 0.67 (PLTMax hPL), 5.6 × 10^6^ ± 0.55 and 5.5 × 10^6^ ± 0.65 (MSC NutriStem XF), 5.1 × 10^6^ ± 1.24 and 5.3 × 10^6^ ± 0.71 (StemXVivo SFM) respectively compared to BM-MSCs cultured in control medium (6.95 × 10^6^ ± 0.63 and 5.45 × 10^6^ ± 0.77) (Fig. 3a). The viable count of BM-MSCs at P5 with seeding densities of 1000 cells/cm^2^ and 5000 cells/cm^2^ was 44.05 × 10^6^ ± 2.47 and 40.22 × 10^6^ ± 1.7 (RoosterNourish), 32.6 × 10^6^ ± 3.32 and 31.6 × 10^6^ ± 0.92 (RoosterNourish-MSC XF), 21.2 × 10^6^ ± 2.68 and 29.1 × 10^6^ ± 2.26 (StemMACS-MSC XF), 33.8 × 10^6^ ± 1.91 and 30.6 × 10^6^ ± 2.35 (PLTMax hPL), 35.6 × 10^6^ ± 1.55 and 28.2 × 10^6^ ± 2.24 (MSC NutriStem XF), 4.8 × 10^6^ ± 0.7 and 1.91 × 10^6^ ± 0.16 (StemXVivo SFM) respectively as compared to cells cultured in control medium (34.8 × 10^6^ ± 2.19 and 32.2 × 10^6^ ± 3.25) (Fig. 3c). The viability of the BM-MSCs was greater than 85% at both P4 and P5 in all media tested (Fig. 3b,d) except for StemXVivo SFM at P5 being 65.1% and 60% at seeding densities of 1000 cells/cm^2^ and 5000 cells/cm^2^ respectively (Fig. 3d). Overall, the cell yield and viability were comparable to the control medium except for MSCs cultured in StemXVivo SFM at P5.

**Figure 4:**
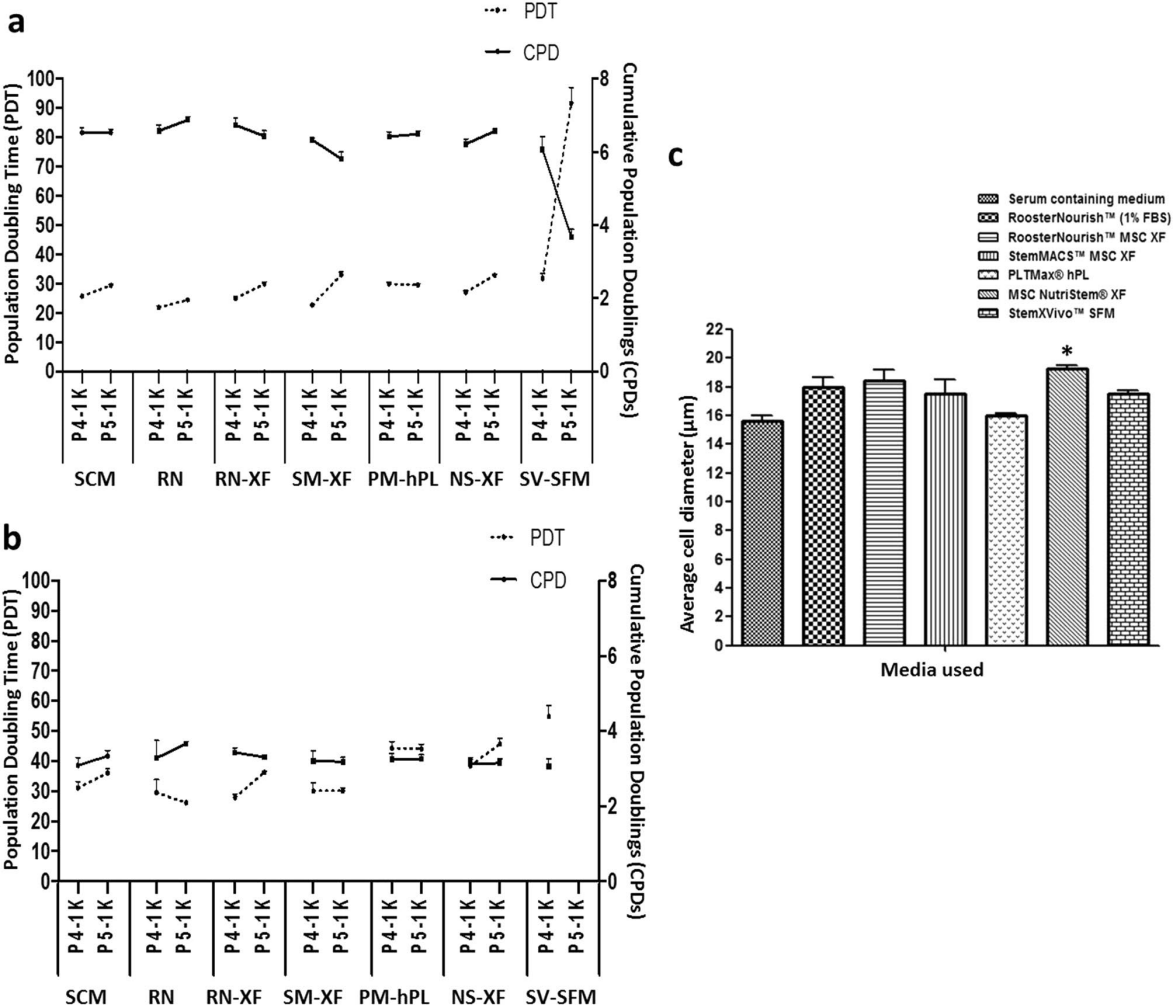
Population doublings and doubling time of BM-MSCs grown in low serum/SFM/XFM and control medium. (**a**) Comparison of PDT and CPDs of BM-MSCs cultured in low serum/SFM/XFM and control medium at P4 and P5 at seeding density of 1000 cells/cm^2^ (**b**) 5000 cells/cm^2^. MSCs cultured in SFM/XFM at 1000 cells/cm^2^ had higher CPDs than MSCs cultured at 5000 cells/cm^2^. BM-MSCs cultured in low serum/SFM/XFM showed varied PDT ranging from 21.89 ± 0.5 h to 45.7 ± 1.66 h except for StemXVivo SFM. **c)** The average cell diameter of BM-MSCs cultured in different low serum/SFM/XFM and control medium analyzed through Vi-CELL XR Cell Viability Analyzer. The average diameter of the BM-MSCs cultured in low serum/SFM/XFM ranged from 16.02 µm ± 0.16 to 19.2 µm ± 0.22. The data is represented as mean ± SD (n = 2*p < 0.05). SCM: Serum containing medium, RN: RoosterNourish, RN-XF: RoosterNourish-MSC XF, SM-XF: StemMACS-MSC XF, PM-hPL: PLTMax human platelet lysate, NS-XF: MSC NutriStem XF, SV-SFM: StemXVivo SFM.

To determine the cell growth kinetics, the PD and PDT of BM-MSCs propagated from P3 to P5 in the respective media was calculated and are shown in Fig. 4a & Fig. 4b. The results showed that BM-MSCs seeded at a density of 1000 cells/cm^2^ had higher proliferation capacity than those seeded at 5000 cells/cm^2^. The cumulative population doubling (CPD) was calculated to compare the proliferation ability of BM-MSCs cultured in these media as opposed to control medium at 1000 and 5000 cells/cm^2^ seeding densities. CPDs (P4-P5) of MSCs grown in RoosterNourish, RoosterNourish-MSC XF, StemMACS-MSC XF, PLTMax hPL, MSC NutriStem XF and StemXVivo SFM at 1000 cells/cm^2^ was 13.44 ± 0.23, 13.16 ± 0.33, 12.14 ± 0.22, 12.91 ± 0.2, 12.77 ± 0.2 and 9.93 ± 0.56 respectively and was comparable with that of control medium (13.06 ± 0.22) (Fig. 4a). Likewise, the CPD (P4-P5) of MSCs grown in RoosterNourish, RoosterNourish-MSC XF, StemMACS-MSC XF, PLTMax hPL, MSC NutriStem XF and StemXVivo SFM at 5000 cells/cm^2^ was 6.94 ± 0.53, 6.74 ± 0.16, 6.39 ± 0.38, 6.51 ± 0.27, 6.26 ± 0.28 and 2.33 ± 0.31 respectively and was comparable with that of control medium (6.42 ± 0.34) (Fig. 4b). However, the CPD of MSCs grown in StemXVivo SFM was significantly lesser (*p* < 0.01) when compared to control medium at both seeding densities (9.93 and 2.33 respectively) since this media was unable to support the growth of MSCs at P5. The PDT increased with the passage number from P4 to P5 in all media evaluated, irrespective of seeding density, as shown in Fig. 4a,b. The PDTs (P4–P5) of BM-MSCs cultured in RoosterNourish, RoosterNourish-MSC XF, StemMACS-MSC XF, PLTMax hPL, MSC NutriStem XF and StemXVivo SFM at 1000 cells/cm^2^ from P4 to P5 were found to be 46.32 ± 0.79 h, 54.77 ± 1.38 h, 55.71 ± 1.19 h, 59.38 ± 0.96 h, 59.8 ± 0.93 h and 123.4 ± 7.17 h respectively compared to that of control medium (55.11 ± 0.92 h) (Fig. 4a). Similarly, the PDT of MSCs grown in RoosterNourish, RoosterNourish-MSC XF, StemMACS-MSC XF, PLTMax hPL, and MSC NutriStem XF at 5000 cells/cm^2^ was 55.79 ± 4.7 h, 64.15 ± 1.5 h, 60.2 ± 3.66 h, 88.48 ± 3.68 h and 84.25 ± 3.74 h respectively compared to that of control medium (67.1 ± 3.64 h) (Fig. 4b). On the other hand, PDT of MSCs cultured in StemXVivo SFM was significantly higher than any other media tested (*p* < 0.05). StemXVivo SFM supported the BM-MSC culture at initial exposure to SFM at P4, but the cells did not proliferate beyond 40% confluence in the subsequent passage. Collectively, the CPDs (P4-P5) of BM-MSCs grown in low serum/SFM/XFM at 1000 cells/cm^2^ were in the range of 5.8–6.7 h and PDT was 21.89–32.88 h. The CPDs at 5000 cells/cm^2^ was in the range of 3.06–3.66 h and PDT was 26.2–45.8 h except for StemXVivo SFM which showed significantly higher PDT and lower CPD. The average diameter of the BM-MSCs cultured in these media was compared and it was observed that the BM-MSCs grown in low serum/SFM/XFM was 18 µm ± 0.65 (RoosterNourish), 18.41 µm ± 0.76 (RoosterNourish-MSC XF), 17.54 µm ± 0.99 (StemMACS-MSC XF), 16.02 µm ± 0.16 (PLTMax hPL), 19.25 µm ± 0.22 (MSC NutriStem XF), 17.53 µm ± 0.2 (StemXVivo SFM) compared to average diameter of 15.63 µm ± 0.36 of BM-MSCs grown in control medium (Fig. 4c). A significant difference was observed in the cell diameter when cultured in MSC NutriStem XF compared to the control medium (p < 0.03).

### Immunophenotype of BM-MSCs cultured in serum-free/xeno-free media before and after cryopreservation

**Figure 5:**
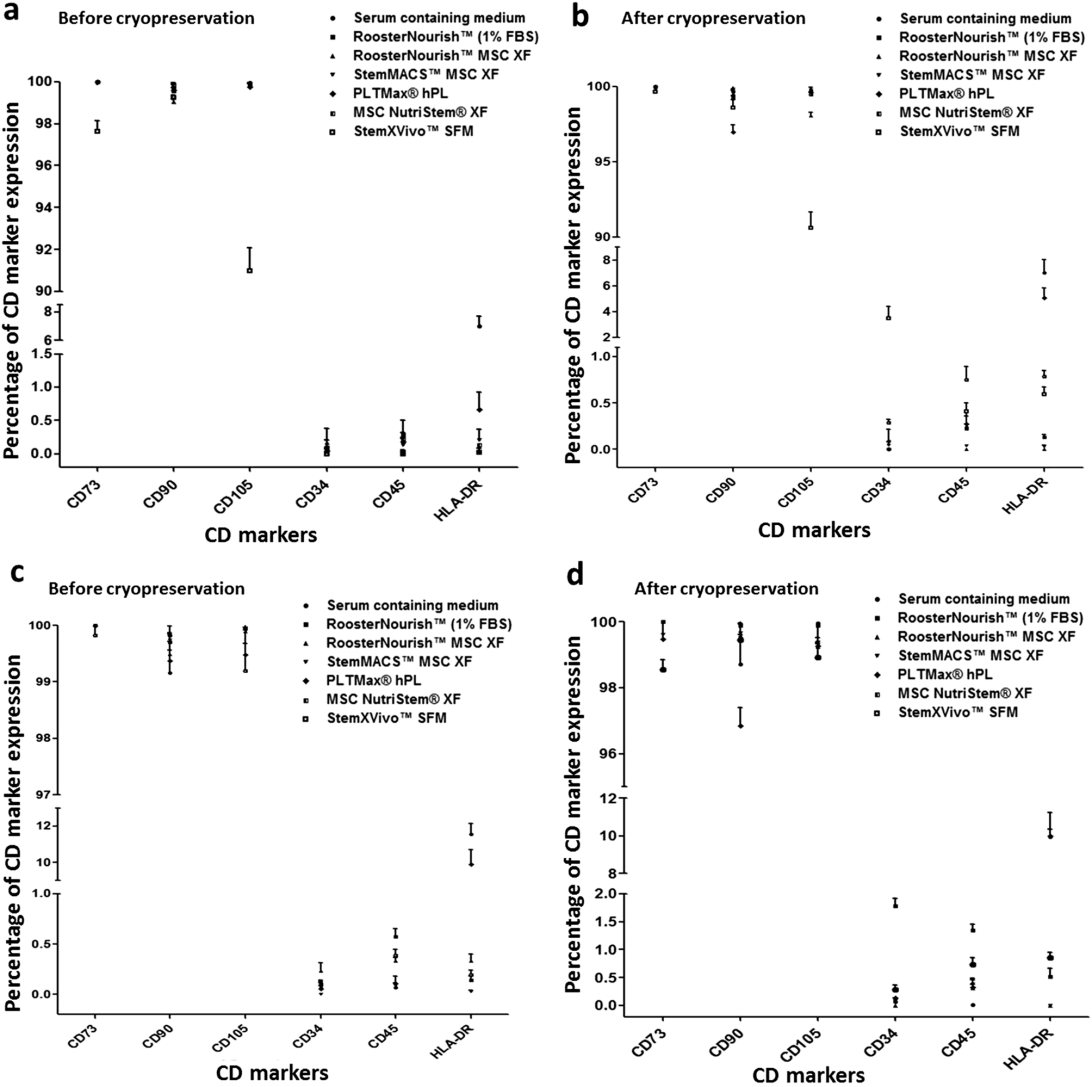
Immunophenotyping of human BM-MSCs cultured in low serum/ SFM/XFM before and after cryopreservation in the two different seeding densities (1000 cells/cm^2^ and 5000 cells/cm^2^). The known positive and negative human MSC specific cell surface antigens were estimated by flow cytometry. Isotope-matched controls were used to determine the non-specific binding by PE and FITC-conjugated antibodies. All the experiments were performed in duplicates and the data is represented as mean ± SD. The expression of positive markers (CD73, CD90 and CD 105) was more than 90% and that of negative markers (CD34, CD45) was less than 5% before and after cryopreservation in the seeding densities of 1000 cells/cm^2^ **(a & b)** and 5000 cells/cm^2^ **(c & d)**. However the expression of HLA-DR of BM-MSCs cultured in control medium and PLTMax hPL were 7.0–11.76% ± 0.66 and 0.67–10% ± 0.76 respectively.

The immunophenotypes of BM-MSCs cultured in low serum/ SFM/XFM and control medium at two different seeding densities were analyzed using flow cytometry before and after cryopreservation (n = 2). BM-MSCs showed good expression of CD markers during adaptation to serum-free conditions. The CD marker expression analysis showed low levels (< 5%) of typical MSC negative markers such as CD34, CD45, and HLA-DR and high levels (> 90%) of positive markers such as CD73, CD90, CD105 for MSCs seeded at 1000 cells/cm^2^ (Fig. 5a & Fig. 5b) and < 10% of negative markers and > 90% of positive markers for MSCs seeded at 5000 cells/cm^2^ both before and after cryopreservation (Fig. 5c & Fig. 5d). BM-MSCs showed comparable expression profile of CD markers except for cells cultured in control medium and PLTMax hPL which showed expression of HLA-DR of 7.0–11.76% and 0.67–10% respectively than any of the media tested. RoosterNourish, RoosterNourish-MSC XF, StemMACS-MSC XF, MSC NutriStem XF showed lowest expression of negative markers (< 1%) and highest expression of positive markers (> 98%) both before and after cryopreservation irrespective of the seeding densities.

### Differentiation potential of BM-MSCs expanded in serum-free/xeno-free media

**Figure 6:**
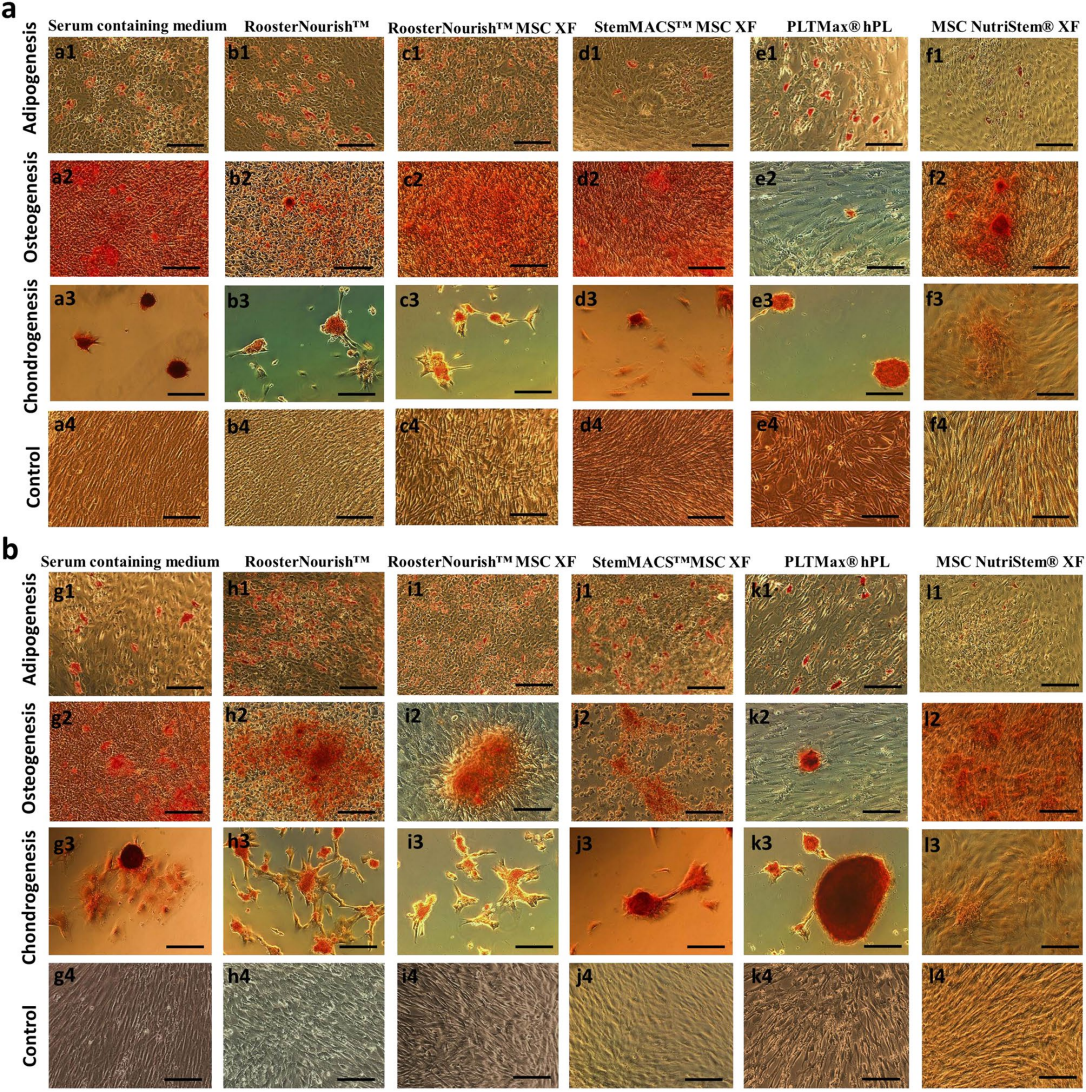
Differentiation potential of BM-MSCs cultured in vitro in low serum/SFM/XFM and control medium during freeze thaw analysis. BM-MSCs cultured at seeding density of 1000 cells/cm^2^ and 5000 cells/cm^2^ was cryopreserved. Freeze thaw analysis was carried out wherein the cells were thawed, revived and seeded to test their ability for trilineage differentiation. (**a**) BM-MSCs grown in SFM/XFM (1000 cells/cm^2^) consistently differentiated into adipogenic (**a1-f1**), osteogenic (**a2-f2**) and chondrogenic (**a3-f3**) lineages, non-induced controls (**a4-f4**). **b)** BM-MSCs grown in SFM/XFM (5000 cells/cm^2^) SFM differentiated into adipogenic (**h1-l1**), osteogenic (**h2-l2**) and chondrogenic (**h3-l3**) lineages, non-induced controls (**h4-l4**) except for BM-MSCs cultured in MSC NutriStem XF. Adipogenic differentiation was detected by oil droplet formation, (oil red O staining). Osteogenic differentiation indicated by calcium accumulation (alizarin red staining). Chondrogenic differentiation detected by Safranin O staining of glycosaminoglycan of cartilage. The images were captured at 10X magnification with scale bar of ∼100 μm.

**Figure 7:**
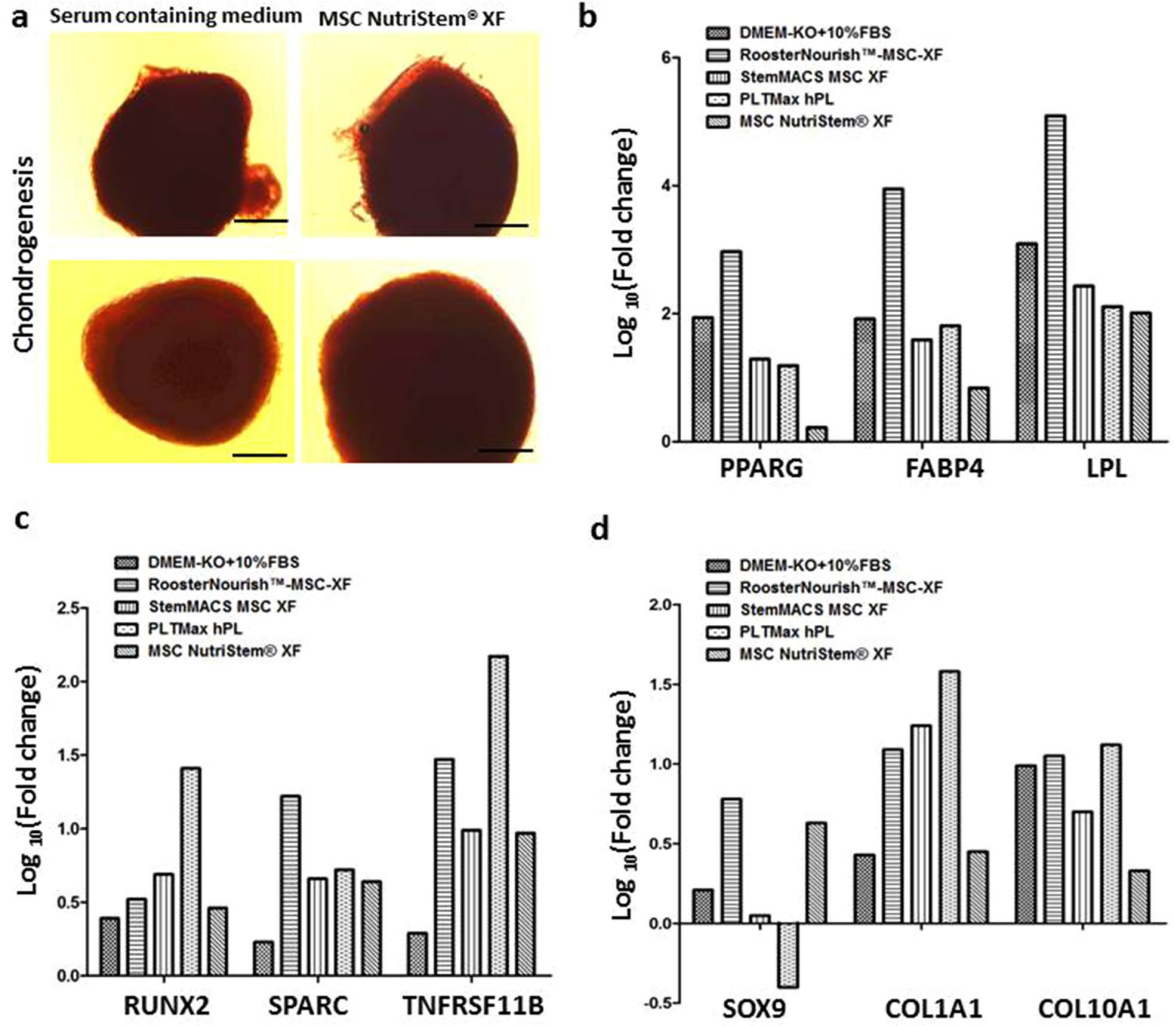
Chondrogenic differentiation and quantification of gene expression in the adipogenic, osteogenic and chondrogenic differentiated cells cultured in SFM/XFM and control medium. (**a**) Chondrogenic differentiation of BM-MSCs in MSC Nutristem XF in 96 well V- bottom plates as evident by 1% Safranin O staining. BM-MSCs cultured in serum containing medium was used as control. The images were captured at 10X magnification with scale bar of ∼100 μm. (**b**) Bar graph showing the fold change in the mRNA expression of *PPARG*, *FABP4* and *LPL* genes in adipogenically differentiated cells relative to the uninduced control (undifferentiated) by real time PCR analysis. (**c**) Bar graph depicts the fold change in the mRNA expression of *RUNX2*, *SPARC* and *TNFRSF11B* genes in osteogenically differentiated cells relative to the uninduced control (undifferentiated). (**d**) Bar graph showing the fold change in the mRNA expression of *SOX9*, *COL1A1* and *COL10A1* genes in chondrogenically differentiated cells relative to the uninduced control (undifferentiated). The Y axis is represented as log_10_ fold change. The log_10_ fold change of uninduced controls is zero. The expression of these genes varied with one another among each of the media tested.

To determine whether low serum/serum-free conditions have any effect on the functional tri-lineage potential of MSCs, we performed an in vitro differentiation assay wherein the cryopreserved cells cultured at seeding densities of 1000 cells/cm^2^ and 5000 cells/cm^2^ were induced to differentiate to adipocytes, osteoblasts and chondrocytes. The differentiation into adipocytes was observed after 3–6 days of induction by Oil red O staining (Fig. 6a: a1-f1 & Fig. 6B: g1-l1) of the fatty vacuole deposits. The differentiation into osteoblasts was detected after 4–6 days of induction by Alizarin red S staining of calcium deposits (Fig. 6a: a2-f2 & Fig. 6b: g2-l2).The differentiation towards chondrocytes was observed after 4–6 days of induction by Safranin O staining (Fig. 6a: a3-f3 & Fig. 6b: g3-l3) with glycosaminoglycan of cartilage. The characteristics of the calcium deposits, fatty vacuole deposits were evident during differentiation of MSCs cultured in RoosterNourish, RoosterNourish-MSC XF, StemMACS-MSC XF, PLTMax hPL, MSC NutriStem XF and was comparable to the control medium; whereas the MSCs grown in MSC NutriStem XF when seeded in 6 well plates were unable to differentiate into chondrocytes. This may be due to cells unable to form micromass when seeded in 6 well plates which require coating with ECM substrate for the attachment of cells (Fig. 6f3 & l3). To overcome this, chondrogenic micromass pellets were generated by seeding BM-MSCs (2 × 10^5^ cells/well) in 96-well V-bottom plates along with the control medium. Differentiation towards the chondrogenic lineage was observed after 14 days of induction as evident by Safranin O staining (Fig. 7a). Overall, BM-MSCs cultured in low serum/SFM/XFM exhibited comparable tri-lineage differentiation potential as evident by their staining characteristics.

To determine the impact of the various SFM/XFM under investigation on the differentiation potential, the expression patterns of key differentiation-specific genes were analyzed in MSC’s which were subjected to adipogenic, osteogenic and chondrogenic differentiation. There was no significant difference in the cellular properties observed when the cells were cultured at seeding densities of 1000 cells/cm^2^ and 5000 cells/cm^2^, hence for further assays cells seeded at 1000 cells/cm^2^ was considered. Early commitment factors and late-stage maturation markers were chosen for analysis. As shown in Fig. 7b, cells cultured in SFM/XFM under evaluation successfully showed adipogenic differentiation. Cells cultured in RoosterNourish-MSC XF exhibited the highest adipogenic differentiation potential compared to other media. With respect to osteogenic differentiation, cells cultured in PLTMax hPL, and RoosterNourish-MSC XF exhibited higher induction of both early (*RUNX2*) and late stage (*SPARC* and *TNFRSF11B*) osteogenic genes (Fig. 7c). Similar results were obtained for chondrogenic differentiation, wherein cells cultured in PLTMax hPL and RoosterNourish-MSC XF showed increased expression of *COL1A1* and *COL10A1* (late- stage maturation genes). However, this did not correlate with enhanced upregulation of the chondrogenic commitment factor *SOX9* (Fig. 7d). Overall assessment of prototypic early commitment and late-stage genes associated with trilineage differentiation indicated better differentiation induction when MSC’s were cultured in RoosterNourish-MSC XF and PLTMax hPL as compared to MSC’s cultured in control medium.

### Colony forming ability of BM-MSCs expanded in serum-free/xeno-free media

**Figure 8:**
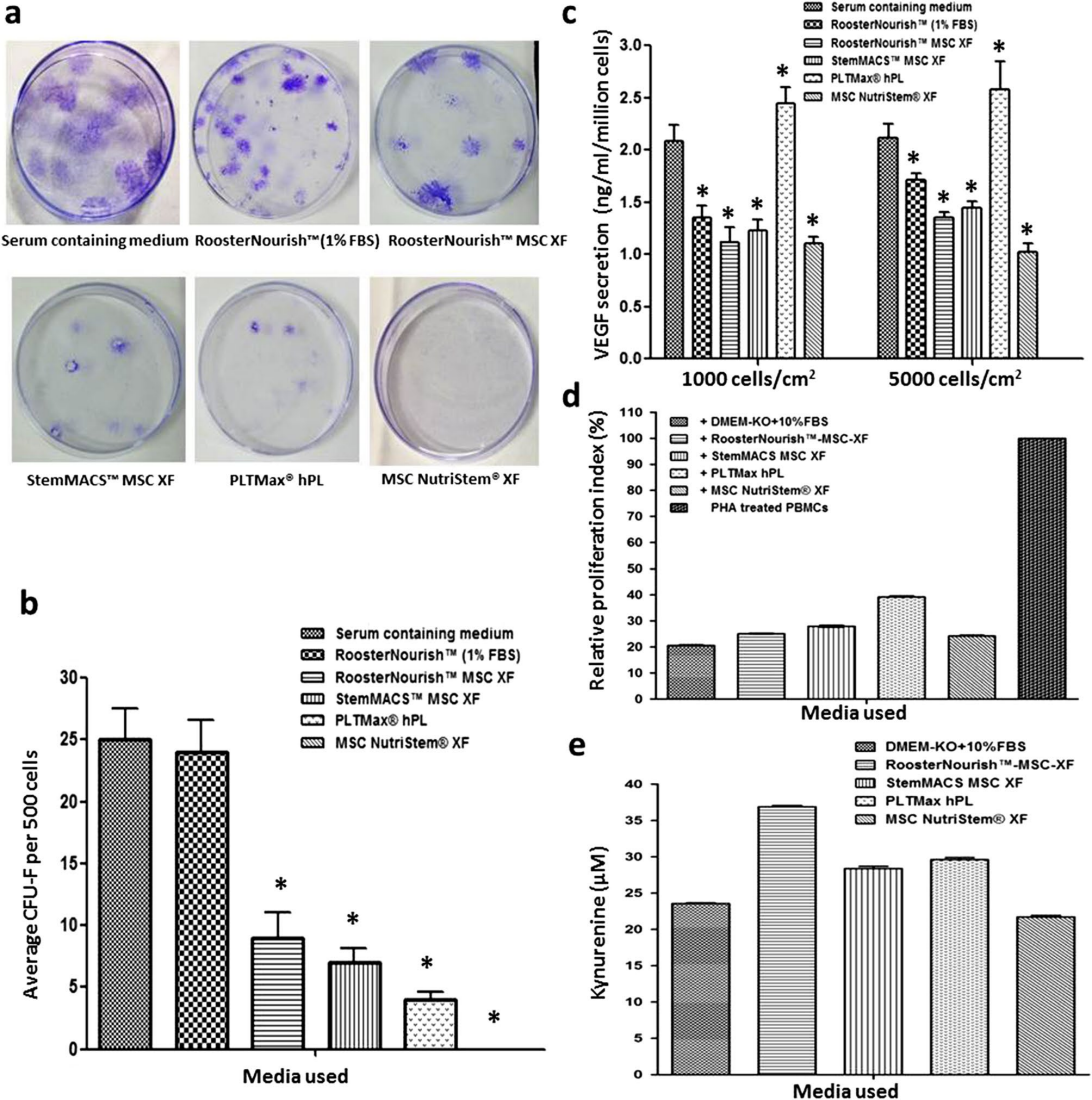
Colony forming ability, potency and immunosuppressive potential of BM-MSCs cultured in SFM/XFM and control medium. (**a**) Representative photograph of CFU-F of BM-MSCs grown in low serum/ SFM/XFM and control medium. (**b**) Bar graph showing the number of colonies of BM-MSCs cultured in low serum/SFM/XFM and control medium. The data is represented as mean ± SD (n = 2; *p < 0.05). (**c**) Cryopreserved cells were seeded into T-75 flasks post revival and grown in respective media for 72 h and the amount of VEGF secreted was determined by ELISA. Results are expressed in concentration (ng/ml/million cells) as mean of duplicate experiment. The amount of VEGF secretion was higher in BM-MSCs cultured in PLTMax hPL when compared to other media and control medium. (**d**) Bar graph shows the percentage proliferation of PHA stimulated T cell blasts in MSC: PHA activated PBMC co-cultures cultured in SFM/XFM and control medium. The proliferation index in co-cultures is relative to proliferation in PHA blasts (cultured in the absence of MSCs) which was considered as 100% (**e**) To determine IDO enzyme activity, the culture supernatant of MSC: PHA activated PBMC co-cultures of different media groups was assayed by spectrophotometric detection of the kynurenine concentration (tryptophan metabolite which is a product of IDO catabolism) and is represented as Kynuerurine units (μM). IDO secretion was higher in co-cultures cultured in SFM/XFM when compared to serum-containing medium.

The colony-forming ability of BM- MSCs cultivated in low serum/ SFM/XFM was tested by CFU-F assay using cells seeded at 1000 cells/cm^2^ post cryopreservation. Typically, MSCs are characterized by their properties of plastic adherence and formation of colonies when plated at low cell densities as determined by CFU-F assay where more than 50 cells are considered as one colony. The CFU-F observed during the culture in various media showed considerable differences in colony morphology. Morphology and number of CFU-F in StemMACS-MSC XF, PLTMax hPL were smaller, few, and dispersed. MSC colonies in RoosterNourish, RoosterNourish-MSC XF were large, varied in shape with more number of colonies. The colonies in the control medium were more in number, larger, and merged (Fig. 8a). The mean CFU-F of cryopreserved MSCs post revival (P6) was 24 ± 2.64, 9 ± 2.08, 7 ± 1.15, 4 ± 0.6 for RoosterNourish, RoosterNourish-MSC XF, StemMACS-MSC XF and PLTMax hPL respectively when compared to control medium (25 ± 2.52) as shown in Fig. 8b. BM-MSCs in MSC NutriStem XF did not show any colonies even after 21 days of culture. The colony forming ability of MSCs cultured in SFM was significantly reduced when compared to the control and RoosterNourish medium (*p* < 0.008).

### Level of VEGF secretion of BM-MSCs cultured in serum-free/xeno-free media

Growth factor/cytokine secretion is one of the characteristics of MSCs that plays a crucial role in cell engraftment, neovascularization, and wound healing and which may be directly linked to the potency of MSCs. One such growth factor is VEGF, which when present at a high level improves the therapeutic efficacy of MSCs. To determine the potency of MSCs grown in the media tested, the amount of VEGF secreted by MSCs when cultured for 72 h post cryopreservation was measured. The level of VEGF secreted by MSCs cultured in RoosterNourish, RoosterNourish-MSC XF, StemMACS-MSC XF, PLTMax hPL and MSC NutriStem XF at 1000 cells/cm^2^ was 1.35 ± 0.12, 1.12 ± 0.13, 1.23 ± 0.1, 2.44 ± 0.15, 1.1 ± 0.07 respectively compared to control medium (2.08 ± 0.15) (Fig. 8c). Likewise, amount of VEGF secreted by MSCs cultured in RoosterNourish, RoosterNourish-MSC XF, StemMACS-MSC XF, PLTMax hPL and MSC NutriStem XF at 5000 cells/cm^2^ was 1.71 ± 0.06, 1.35 ± 0.05, 1.44 ± 0.06, 2.58 ± 0.26 and 1.02 ± 0.08 respectively compared to control medium (2.11 ± 0.07) (Fig. 8c). The amount of VEGF secreted by MSCs cultured in RoosterNourish, RoosterNourish-MSC XF, StemMACS-MSC XF and MSC NutriStem XF was significantly less when compared to control medium whereas PLTMax hPL secreted higher amount of VEGF than control medium. Taken together, MSCs cultured in PLTMax hPL secreted significantly higher amounts of VEGF when compared to other media and control medium (*p* < 0.01).

### BM-MSCs cultured in serum-free/xeno-free media retain immune suppression ability

Apart from their trilineage differentiation potential, the essential feature that makes MSC’s useful for clinical applications is their immunomodulatory ability. The immunomodulatory property of MSC’s was studied using an in vitro Mitogen induced lymphoproliferation assay wherein MSC’s ability to suppress phytohaemagglutinin (PHA) stimulated T cell blast formation was investigated. As shown in Fig. 8d, cells cultured in SFM/XFM retained the ability to suppress T cell blast formation. More than 60% T cell blast suppression was noted in co-cultures of MSCs cultured in all the SFM/XFM tested.

### Serum-free/xeno-free media cultured MSCs exhibit indoleamine 2,3 dioxygenase (IDO) induction in T-cell blast co-cultures indicating conservation of key mechanisms of immune-suppression

Secretions of IDO have been reported as one of the key mechanisms underlying human MSC mediated suppression of T cell response^36^. IDO secretion is induced in human MSCs by IFN-γ present in the pro-inflammatory milieu. IDO depletes tryptophan, an essential amino acid in the local environment required for T cell proliferation and survival. BM-MSCs cultured in SFM/XFM were tested for their ability to secrete IDO in MSCs: PHA activated PBMC co-cultures. As shown in Fig. 8e, cells cultured in SFM/XFM showed substantial IDO activity induction in the pro-inflammatory environment existing in the co-cultures. However, MSCs cultured in RoosterNourish-MSC XF secreted higher IDO in co-cultures compared to MSCs cultured in other media. Nonetheless IDO induction remains one of the probable mechanisms of immunosuppression in all the media tested.

## Discussion

MSCs isolated from different tissues are generally expanded in vitro using animal-derived growth serum (FBS). However, the use of MSCs cultured in FBS containing media for clinical applications poses potential risk and safety concerns due to the possibility of exposure to infectious agents and animal antigen^33^. Lot-to-lot variability and cost-intensive pre-testing of FBS samples raise concern for its usage in stem cell therapy. A few studies have employed a defined low-serum containing medium for MSC growth demonstrating efficient proliferation and maintenance of MSC characteristics leading to long-term consistency of results^37,38^. To eliminate the use of animal-derived growth supplements, several research laboratories have developed “in-house” SFM for MSC culture with a wide range of media supplements and culture ingredients and investigated MSC properties with variable results^21,39–43^. However, the biosafety of the media components in these disclosed formulations is not ensured and warrants further analysis. Moreover, some of the studies have shown differences in the growth of MSCs derived from different tissues of the same species^16,28,31^ or same tissue of different species when cultured in defined SFM indicating serum-free culture conditions are tissue and species-specific and underlines the need for optimization for MSCs from different origin and/or relevant animal species^30,44^. To overcome these limitations, proprietary serum/xeno-free media, which are commercially available, have been developed as potential alternatives for in vitro clinical-grade MSCs expansion. There have been limited comparative studies with mixed results investigating the performance of these media and their effect on the properties of MSCs derived from different sources^27,33,45–47^. Few studies have assessed these media for MSC characterization and have shown their suitability for MSC growth and expansion while adhering to the minimal criteria set by the International Society of Cellular Therapy^14,16,33,48–53^. However, there is no comprehensive study available evaluating the suitability of these media in supporting the growth of BM-MSCs initially cultured under serum conditions. Hence, the present study was undertaken to investigate the use of six different commercially available media for BM-MSC expansion and compared the MSC culture characteristics and functions with serum-containing medium. As new generations of SFM are emerging from various companies, these data may be substantial to address the specific challenges associated with the expansion of MSCs for therapeutic use during the transition from serum-containing media to serum-free media.

The novelty of this study compared to earlier studies is that: (1) this is a comprehensive study evaluating six different commercially available low serum/SFM/XFM for its suitability in supporting the growth and expansion of BM-MSCs initially cultured under serum conditions until passage 3. More interestingly, the study provides experimental evidences and baseline data to support the growth and expansion of BM-MSCs using SFM/XFM from the cell banks that are already created using serum containing media without additional evaluation to test the suitability of the medium, (2) two different seeding densities of MSCs have been evaluated to determine the suitability of the medium to support growth and expansion of BM-MSCs. (3) BM-MSCs used in the present study were produced by pooling cells from 3 different donors at passage 2, which reduces individual variability in properties such as rate of proliferation, secretory activity, and differentiation, resulting in a product with robust paracrine activity.

Earlier studies have reported that the lower seeding density (50–1000 cells/cm^2^) supports the efficient proliferation of MSC cultures in serum media^54^. Nevertheless, the seeding densities for MSC cultures must be optimized to minimize growth in patches observed when the seeding density is below the suboptimal range^6^. Abrahamsen et al., (2002) reported that lower seeding densities are perhaps better for expansion of MSCs since less contact inhibition is observed which is regulated by the Wnt signaling pathway^55^. In the present study, we carried out the comprehensive characterization of six different commercially available media through the passive adaptation method of frozen cultures, wherein the BM-MSCs were isolated and cultured in the serum-containing medium until passage 3 was subsequently cultured in low serum/SFM/XFM for the next 2 passages with seeding densities of 1000 cells/cm^2^ and 5000 cells/cm^2^. BM-MSCs showed similar proliferation potential, expansion efficiency, and cell viability (> 85%) in RoosterNourish, RoosterNourish-MSC XF, PLTMax hPL, StemMACS-MSC XF and MSC NutriStem XF when compared to control medium at both seeding densities and were chosen for further testing to determine the functional characteristics. Although StemXVivo SFM showed comparable cell numbers at P4, it was unable to support growth at P5 at both seeding densities. MSCs cultured with the seeding densities of 1000 cells/cm^2^ and 5000 cells/cm^2^ demonstrated almost equivalent cell yield and viability, surface marker expression, and tri-lineage differentiation. These results suggested that even at a lower seeding density of 1000 cells/cm^2^, MSCs were able to proliferate efficiently in SFM while maintaining its functional characteristics.

The maintenance of a steady PDT is crucial for the expansion of MSCs for therapeutic use. Most of the serum-free formulations tested claim equivalent cell population doubling time and preservation of MSC characteristics when compared with serum-containing medium^22,51,56,57^. Growth kinetics of BM-MSCs in SFM evaluated in the present study showed varied PDT ranging from 21.89 to 45.7 h except for StemXVivo SFM and this is in concurrence with previous studies reporting similar PDT of MSCs cultured in SFM^26,28,31,48^ . However, the performance with each of the medium varied with one another and with control medium which may be attributed to the differences in the composition of the medium^45,58,59^. Notably, BM-MSCs grown in RoosterNourish, RoosterNourish-MSC XF, StemXVivo SFM, StemMACS-MSC XF and MSC NutriStem XF seemed to be similar in size to one another with an average cell diameter higher than control medium, while PLTMax hPL cultured BM-MSCs displayed average cell diameter similar to control medium. The BM-MSCs grown in SFM appeared larger and thicker when compared to the serum containing medium. Whether these morphological characteristics are related to cell expansion capacity is elusive.

Immunophenotype analysis before and after cryopreservation revealed that BM-MSCs cultured in SFM expressed higher MSC specific surface markers CD73, CD90, and CD105, whereas their expression levels of hematopoietic cell markers (CD34, CD45) and HLA-DR molecules were very low satisfying the phenotypic criteria for describing hMSCs^60^. One interesting observation is the expression of HLA-DR in RoosterNourish, RoosterNourish-MSC XF, StemXVivo SFM, StemMACS-MSC XF and MSC NutriStem XF cultured BM- MSCs was low compared to that of PLTMax hPL and serum-supplemented cultures. A few studies have shown that BM-MSCs cultured in serum-supplemented medium with bFGF tend to express HLA-DR^54,61^. In this direction, serum-free conditions would be safer for large-scale manufacturing leading to lower HLA-DR expression.

To assess the multipotentiality of BM-MSCs grown in low serum/SFM/XFM, cryopreserved cells were induced to differentiate into trimesenchymal lineages, and relative expression of the representative osteogenic, chondrogenic and adipogenic genes were quantified. MSCs cultured in different SFM/XFM exhibited trilineage differentiation potential, albeit to varying levels. BM-MSCs cultured in RoosterNourish and RoosterNourish-MSC XF certainly differentiated towards trimesenchymal lineages and was comparable to the serum- containing medium. Overall, higher level of differentiation induction was observed when MSC’s were cultured in RoosterNourish-MSC XF and PLTMax hPL compared to MSC’s cultured in other media. Furthermore, to evaluate the potential of cells expanded in SFM, human MSCs cultured in low serum/SFM/XFM were placed into a CFU-F assay. The CFU-F efficiency of BM-MSCs cultured in SFM/XFM media was significantly less when compared to the control medium, whereas colony-forming ability of BM-MSCs grown in RoosterNourish (containing 1% FBS) was comparable to the control medium.

Recent studies have detailed the importance of determining the potency of human MSCs to predict their therapeutic efficacy and assessing the paracrine factors and immunomodulatory status of MSCs to develop better standards^62–64^. Moreover, it is reported that MSC potency may be directly linked to their secretory profile^65,66^. VEGF is one such factor that is secreted by MSCs and is essential for osteogenic differentiation and angiogenesis^67^. Our previous research findings established VEGF as a surrogate potency marker for BM-MSCs and through the VEGF quantitation data obtained from a large number of production batches; we estimated the level of VEGF ≥ 2 ng/ml/million BMMSCs to qualify for producing angiogenic activity. Also, we established a high correlation of VEGF levels with in vitro endothelial function^68^. In the present study, the level of VEGF secreted by cryopreserved MSCs cultured in different media was estimated. MSCs cultured in PLTMax hPL secreted a higher amount of VEGF (> 2 ng/ml/million cells); while MSCs cultured in other media secreted VEGF < 2 ng/ml/million cells. The reason for the lower level of VEGF secretion by MSCs when cultured in SFM needs to be further investigated. Furthermore, BM-MSCs cultured in SFM/XFM retained their immunosuppressive potential. Several secretory molecules have been proposed to play a crucial role in regulating the immunosuppressive effects of MSCs, including indoleamine 2,3-dioxygenase, nitric oxide, prostaglandin E2, transforming growth factor beta and so on^34^. Besides, we quantified the levels of IDO secretion in BM-MSCs and it was observed that cells cultured in SFM/XFM secreted significantly higher level of IDO compared to the control medium. Immunosuppression activity is a multifactorial process; hence, the exact mechanism through which the SFM/XFM grown cells render immunosuppression remains to be determined.

In conclusion, our research findings successfully demonstrated the growth of pre-isolated MSCs (P3) in low serum/serum-free conditions for two subsequent passages at the lower seeding density of 1000 cells/cm^2^ while retaining their functional properties. This is the first comprehensive study reporting the extensive characterization of BM-MSCs in six different commercially available low serum/SFM/XFM along with serum-containing medium with low and high seeding densities. There was no significant difference in the expression of MSC specific markers among each of the media tested. On the other hand, the cell yield, population doubling time, potency in terms of VEGF secretion, colony forming ability, differentiation potential to mesodermal lineage and immunosuppressive potential of MSCs varied with one another. The present study showed that among the media evaluated, RoosterNourish-MSC XF and PLTMax hPL would be preferable in terms of cell yield, preserving MSC characteristics and reduced overall costs. MSC NutriStem XF and StemXVivo SFM requires extra step of coating the culture surface which may not be feasible during large scale production of MSCs for clinical applications. However, by eliminating the use of FBS it is certain that these SFM tested accomplish the key requirements in terms of multi-passage expansion of MSCs. This study provides useful information for the researchers from different laboratories to conduct studies using suitable SFM without need for additional step of evaluation. The most suitable media for MSC expansion should be chosen accordingly as different media produce MSCs with different properties. Future optimization studies and assessment of cell performance are required in large scale culture system before employing these media for therapeutic applications with the requirement to achieve cGMP and regulatory compliance.

## Materials and methods

### Isolation and culturing of BM-MSCs

Bone marrow was aspirated from healthy donors of age group between 18 and 40 years, after receiving written informed consent and approval in accordance to the Manipal University Ethics Committee Guidelines (Protocol Number: SRPL/CLI/07-08/001). All the experimental protocols were approved by the Institutional Committee for Stem Cell Research. Human mesenchymal stromal cells was isolated by density gradient centrifugation as described previously^69^. Cells were plated in T-75 flasks with DMEM-KO (Thermo Fisher Scientific, USA) containing 10% FBS (Hyclone, Waltham, USA), 2 mM L-Glutamax (Invitrogen, USA) and maintained at 37 °C incubator with 5% humidified CO_2._ Media change was performed to remove non-adherent cells at defined intervals. The MSCs were grown in serum-containing medium up to passage 3 (P3) and are derived from multiple donor pool (3 different donors).

### Sub culturing and expansion of BM-MSCs in different serum-free media

Human MSCs that are cryopreserved at P3 were thawed at 37 °C in a water bath, revived with pre-warmed culture medium, centrifuged at 1200 rpm for 10 min. The pellet obtained was then dislodged, resuspended in DMEM-KO, and seeded (P4) at low (1000 cells/cm^2^) and high seeding densities (5000 cells/cm^2^) in T-75 flasks (Thermo Fisher Scientific, USA) with low serum/SFM/XFM as listed in Table-1 by employing passive adaptation method^45^. The T-75 flasks were coated with MSC attachment solution (Biological Industries, USA) for MSCs cultured in NutriStem XF Medium and human fibronectin (R&D Systems, Minneapolis, USA) for MSCs cultured in StemXVivo human mesenchymal stem cell expansion media according to manufacturer’s instructions. Other SFM tested are coating-free media. The composition of this media is proprietary and is not available publicly. MSCs cultured in DMEM-KO medium supplemented with 10% FBS, 2 mM L-Glutamax, 1X Pen-Strep (Invitrogen, USA) and 2 ng/ml bFGF (Invitrogen, USA) served as the control (hereafter referred as control medium). The cultures were screened at regular intervals to check confluency, morphological, and phenotypic characteristics. Once the cultures attained 80–90% confluency, the cells were harvested by enzymatic digestion with 0.25% trypsin–EDTA (Gibco, USA) at 37 °C for 2–3 min followed by neutralization of trypsin activity. The resulting cell suspension was expanded in the next passage (P5) in CellSTACK—1 (CS-1) chamber with vent caps (Corning, New York, USA) at low and high seeding densities. Media change was performed when the cultures attained 55–60% confluency and were harvested at 80–90% confluence. The cells were washed two times with Dulbecco’s phosphate-buffered saline (DPBS, Gibco, USA) for 2–3 min. The BM-MSCs were trypsinized by addition of pre-warmed 0.25% trypsin–EDTA and incubation for 2–3 min at 37 °C followed by neutralization of trypsin activity by using the respective media. The cells were then centrifuged at 1200 rpm for 10 min, followed by DPBS wash and centrifugation. The cells thus obtained were resuspended in Cryostor 5 freezing media (BioLife Solutions, USA) and filled in 1.8 ml Nunc vials at a density of 5 × 10^6^ cells/ml. The vials were then freezed in a freezing container (Nalgene Mr. Frosty, Sigma, USA) at a cooling rate of 1 °C/min in a − 80 °C freezer. After 12–16 h, the frozen vials were stored in liquid nitrogen below − 150 °C.

### Population doublings

To study the growth kinetics, pre-isolated BM-MSCs (cultured in serum-containing medium up to passage 3) grown in low serum/SFM/XFM from P3 onwards were used. The population doublings (PDs) and doubling time (PDT) of BM-MSCs cultured in these media and control medium were estimated from P3 to P5 and harvesting was carried out after reaching 80–90% confluency in each passage.

### Immunophenotype analysis by flow cytometry

To examine the effect of freezing and thawing on MSC functions, the cryopreserved BM-MSCs (P5) were thawed quickly at 37 °C, revived with Plasmalyte-A (Baxter, USA). The cell count and viability were assessed using Vi-CELL XR Cell Viability Analyzer (Beckman Coulter, USA) which works on trypan blue dye exclusion principle. The resulting cell suspension was centrifuged, resuspensded in respective media and used for analyzing CD marker expression, differentiation potential, potency and ability to form CFU-F. The StemXVivo SFM was unable to support the growth of MSCs at P5 in both the seeding densities tested and hence freeze thaw analysis of MSCs cultured in StemXVivo SFM was not performed. The surface marker expression profile of BM-MSCs was determined by immunophenotyping using flow cytometry. In brief, 200 µl cell suspension was incubated with known concentrations of phycoerythrin (PE) or fluorescein isothiocyanate (FITC)- conjugated antibodies for 30 min in dark at 4 °C. The following CD markers were analyzed: CD34-PE, CD90-PE, CD105-PE (Chemicon), HLA-DR-FITC, CD45-FITC, CD73-PE and (BD Pharmingen, San Diego, CA, USA). To set the background fluorescence levels, appropriate isotype-matched controls were used. Post incubation, cells were washed thrice and resuspended in 0.5 ml wash buffer for the analysis. Flow cytometry was performed using an EasyCyte and analyzed using Guava Express Pro software (Guava Technologies, USA).

### Differentiation potential

The tri-lineage differentiation capacity of BM-MSCs cultured in low serum/SFM/XFM or control medium was evaluated during freeze–thaw analysis. Recovered BM-MSCs (7 × 10^5^ cells) were cultured in the respective media in 37 °C incubator with 5% CO_2_ and seeded into StemPro Osteogenesis, Adipogenesis or Chondrogenesis Differentiation medium (Gibco, USA) in 6-well plates as per the manufacturers protocol. Complete media change was performed during the culture period once in 3 to 4 days till they attain 90% confluency. The cells were then induced for adipogenesis and osteogenesis; screened at regular intervals for differentiation. Once the differentiation is observed, staining of cells was performed with 2% Alizarin red S (Sigma-Aldrich, USA) to monitor osteogenic differentiation and 6.3% Oil Red O (Sigma-Aldrich, USA) for adipogenic differentiation. For chondrogenic differentiation, chondrogenic micromass pellets were generated by seeding 1 × 10^6^ recovered cell suspension in chondrogenic differentiation medium in 6-well plates and/or 2 × 10^5^ cells/well in 96-well V-bottom plates (CELLSTAR, Greiner Bio-One GmbH) after centrifugation at 1200 rpm for 5 min, and placed in an incubator maintained at 37 °C with 5% CO_2_. Medium change was performed once in every 3 days. The cells were screened at regular intervals and once the differentiation was observed, the micromass pellet cultures were stained with 1% Safranin-O (Sigma, USA). The differentiated cells were visualized after fixing with 10% formalin and washing with DPBS. The images were captured using IX-71 inverted microscope (Olympus, Japan) under 10X objective. The uninduced cultures expanded in the respective media served as control.

### Quantitative gene expression analysis

Total RNA was extracted using RNA isoplus (Takara Bio) and cDNA was synthesized using PrimeScript RT-PCR kit (Takara Bio). Gene expression analysis was carried out using SYBR green (TB green Premix Ex Taq II ,Takara Bio) on Step One plus qPCR instrument (Applied Biosystems) using gene specific primers as listed in Table 2. Relative mRNA expression levels were normalized to GAPDH and ΔCt was calculated. ΔΔCt was calculated by subtracting ΔCt of test with ΔCt of the calibrator (uninduced MSC’s). The fold change was calculated using the formula 2^−ΔΔCt^ and represented as log_10_ (fold change) after calculating the mean values of triplicates.

**Table 2:** List of primers used for RT-PCR analysis.

Gene	Primer sequence	Product length
*GAPDH* (Glyceraldehyde-3-Phosphate Dehydrogenase)	Forward: TGGTATCGTGGAAGGACTCATGAC Reverse: ATGCCAGTGAGCTTCCCGTTCAGC	189 bp
*PPARG* (Peroxisome Proliferator Activated Receptor Gamma)	Forward: CCATGCTGTTATGGGTGAAA Reverse: TCAAAGGAGTGGGAGTGGTC	192 bp
*FABP4* (Fatty Acid Binding Protein 4)	Forward: ACCTTAGATGGGGGTGTCCTGGT Reverse: CGCCTTTCATGACGCATTCCACC	112 bp
*LPL* (Lipoprotein Lipase)	Forward: ATGGAGAGCAAAGCCCTGCTC Reverse: GTTAGGTCCAGCTGGATCGAG	564 bp
*RUNX2* (RUNX Family Transcription Factor 2)	Forward: CCACTGAACCAAAAAGAAATCCC Reverse: GAAAACAACACATAGCCAAACGC	129 bp
*PARC* (Secreted Protein Acidic And Cysteine Rich)	Forward: TTCAGCCAGGAAGGCCAAAA Reverse: GGGGAGGGTGAAGAAAAGGA	146 bp
*TNFRSF11B* (TNF Receptor Superfamily Member 11b)	Forward: GTGGAATAGATGTTACCCTGTGTG Reverse: TGCTCGAAGGTGAGGTTAGC	298 bp
*SOX9* (SRY-Box Transcription Factor 9)	Forward: GGCAAGCTCTGGAGACTTCTG Reverse: CCCGTTCTTCACCGACTTCC	138 bp
*COL1A1* (Collagen Type I Alpha 1 Chain)	Forward: AGGGCTCCAACGAGATCGAGATCCG Reverse: TACAGGAAGCAGACAGGGCCAACGTCG	223 bp
*COL10A1* (Collagen Type X Alpha 1 Chain)	Forward: AGCCAGGGTTGCCAGGACCA Reverse: TTTTCCCACTCCAGGAGGGC	386 bp

### Colony forming unit-fibroblast (CFU-F) assay

Recovered BM-MSCs (total of 500 cells) were plated in respective media and control medium (n = 2 per group) in a 60 mm cell culture dish (Thermo Fisher Scientific, USA). The plates were stained with 0.5% crystal violet solution (Sigma, USA) 14 days post incubation and visible colonies were counted. The data was reported as average number of colonies for each group and represented as mean ± SD.

### Vascular endothelial growth factor secretion (Freeze–thaw analysis)

Conditioned medium from BM-MSCs was prepared and the amount of VEGF secreted was estimated as described previously^68^. In brief, the recovered cells (total 1 × 10^6^ cells) were seeded in T-75 flasks in respective low serum/SFM/XFM media and control medium. The cultures were incubated at 37 °C incubator and 5% CO_2_. After 72 h, the conditioned medium was collected; filtered using a 0.22 μm syringe filter (Merck-Millipore, NJ, USA), aliquoted and stored at –80 °C until use.

### Enzyme-linked immunosorbent assay

Human angiogenic factor (VEGF) secreted by MSCs was estimated by enzyme-linked immunosorbent assay (ELISA) using Human Quantikine ELISA kit (R&D Systems, Minneapolis, USA) according to the manufacturer’s protocol. The respective media were used as controls. In brief, 200 μL of conditioned media was added to 96-well microplates that were pre-coated with monoclonal antibody specific for human VEGF and incubated for 2 h. After washing with wash buffer, enzyme-linked polyclonal antibody specific for human VEGF was added to each well, followed by incubation for 2 h and washing to remove unbound antibody-enzyme reagent. The substrate solution was added and incubated for 30 min, and the reaction was terminated by the addition of the stop solution. The level of VEGF was estimated by measuring the optical density at 450 nm using a VersaMax microplate reader (Molecular Devices, USA). The cells were harvested after 72 h by enzymatic digestion using 0.25% Trypsin–EDTA followed by neutralization with respective media. The cell count was estimated using the Vi-CELL XR Cell Viability Analyzer. The samples were assayed in duplicates and the amount of VEGF secreted was represented as ng/ml/ million cells.

### Mitogen induced lymphoproliferation assay and BrdU incorporation assay

BM-MSC’s were seeded at a density of 20 × 10^3^ cells per well of a 96 well plate in their respective media and allowed to adhere overnight. On the subsequent day they were treated with Mitomycin C (10 µg/ml) for 2 h, washed with PBS to remove traces of mitomycin and recovered in their respective media for 24 h. PBMC’s were isolated using lymphoprep (Axis shield) as per manufacturer’s instructions. The buffy coat layer was collected, washed thrice with PBS and then resuspended in respective SFM/XFM except for the control group where the PBMC’s were resuspended in RPMI supplemented with 10% FBS. In order to stimulate T cell blast formation, PHA (20 µg/ml) was added to the PBMC suspension after which 1 × 10^5^ PBMC’s were added to MSC’s to obtain MSC: PBMC ratio of 1:5 as previously optimized. The co-culture was continued for 72 h and BrdU priming was performed 12 h before lymphoproliferation assessment. After 72 h, BrdU incorporation assay (BrdU Cell proliferation kit, Calbiochem) was performed as per the manufacturer’s instructions. PBMC alone, Mitomycin C treated MSC alone and PBMC co-cultured with MSC served as background controls whereas PHA treated PBMC’s served as positive controls. The extent of T cell blast suppression was calculated by determining the level of BrdU incorporation compared to the PHA treated PBMCs which was considered as 100%.

### Indoleamine 2,3 dioxygenase assay

Freshly collected supernatants from co-cultures were treated with half the volume of 30% trichloroacetic acid, vortexed and then centrifuged at 10,000 rpm for 5 min. The resulting supernatant was collected to which equal volume of Ehrlich reagent (100 mg in 5 ml glacial acetic acid) was added, incubated for 10 min at room temperature after which absorbance was measured at 490 nm. IDO activity was assessed by measuring the kynurenine concentration as determined by plotting the absorbance values against the kynurenine standard curve.

### Statistical analysis

Quantitative data were reported as mean ± standard deviation (SD). Analysis of variance (ANOVA) and unpaired t test were performed using GraphPad Prism Software (www.graphpad.com). Results were considered statistically significant if *p* value was < 0.05.

## Data Availability

The datasets generated and analyzed during the current study are available from the corresponding author on reasonable request.
